# Metabolic Obesity in People with Normal Body Weight (MONW)—Review of Diagnostic Criteria

**DOI:** 10.3390/ijerph19020624

**Published:** 2022-01-06

**Authors:** Waldemar Pluta, Wioleta Dudzińska, Anna Lubkowska

**Affiliations:** 1 Department of Functional Diagnostics and Physical Medicine, Pomeranian Medical University in Szczecin, Żołnierska 54, 71-210 Szczecin, Poland; wioleta.dudzinska@pum.edu.pl (W.D.); anna.lubkowska@pum.edu.pl (A.L.); 2 Department of Physiology and Biochemistry, Institute of Biology, University of Szczecin, Felczaka 3c, 71-412 Szczecin, Poland

**Keywords:** MONW, obesity, diagnostic criteria

## Abstract

Disorders of metabolic obesity with normal body weight (MONW) are widely recognized risk factors for the development of cardiovascular diseases and type 2 diabetes. Despite this, MONW is not diagnosed in clinical practice. There is no consensus on the definition of MONW, and measuring the degree of insulin resistance or obesity among apparently healthy, non-obese patients is not widely applicable. The awareness of the relationship between metabolic disorders such as MONW and a higher risk of mortality from cardiovascular causes and other related diseases prompts the need for action to be taken aimed at creating appropriate diagnostic models that will allow for the effective detection of those with metabolic abnormalities among people with normal body weight. Such actions are decisive in the prevention and treatment of diseases. Therefore, the purpose of this article is to review the MONW diagnostic criteria used over the years.

## 1. Introduction

Modern human lifestyle is not conducive to maintaining health. Sedentary work, low physical activity, improper diet, irregular meals and snacking between them, as well as overeating in the evening, promote obesity [1]. According to the definition provided by the World Health Organization (WHO), overweight and obesity are defined as abnormal or excessive fat accumulation that presents a risk to health [2]. Statistics on the percentage of people with excessive adipose tissue are not optimistic. The Global Burden of Disease Group who analyzed data from 68.5 million persons from 195 countries reported in 2017 that between 1980 and 2015, the prevalence of childhood and adult obesity has doubled in 73 countries and shows a steady increase in most other countries [3]. Moreover, the results of Ward et al., suggest that by 2030 every second adult person will have obesity and every fourth adult person will have severe obesity [4].

Obesity is usually caused by supplying the body with too many nutrients in relation to the amount needed. This excess is stored in the body as triglycerides, commonly known as fat, and the adipocytes where triglycerides are stored, are known as fat cells. Increased fat mass can manifest itself by increasing the size of the adipocyte cells (hypertrophy) and proliferation (hyperplasia). When adipocytes cannot uptake excess triglycerides it leads to adipogenesis, creating extra space for large amounts of fat to be stored [5].

Excessive body fat is conducive to the development of many diseases, including: metabolic syndrome (MetS), type 2 diabetes mellitus (T2DM), hypertension, ischemic heart disease, atherosclerosis, hyperlipidemia, non-alcoholic fatty liver, as well as complications related to the osteoarticular, musculoskeletal and respiratory systems. Moreover, obesity is one of the risk factors for breast, uterine, esophageal and kidney cancer [1,6,7].

Obesity is a heterogeneous disorder. People with obesity are characterized by inter-individual variability in terms of the distribution of adipose tissue, metabolic profile and the degree of cardiovascular and metabolic risk. Abdominal fat storage is much more conducive to the development of T2DM and coronary diseases than peripheral or gluteal–femoral obesity. Significant anatomical, cellular, molecular, physiological, clinical and prognostic differences are also observed between subcutaneous adipose tissue (SAT) and visceral adipose tissue (VAT) [8]. Although both types of adipose tissue have been shown to be responsible for the development of insulin resistance [9], the excess of visceral depot may turn out to be detrimental to human health. Visceral adipose tissue is much more metabolically and hormonally active compared to its subcutaneous counterpart, it also exhibits pro-inflammatory properties and is prone to lipolysis. In addition to the aforementioned insulin resistance, visceral adipose tissue plays a significant role in the development of T2DM, glucose intolerance, hypertension and cardiovascular disease [10,11]. Long-term observations showed a significant positive association between increased levels of VAT and an increased risk of cardiovascular disease. No such relationship was observed in the case of SAT [12].

Visceral fat became the subject of interest in the 1980s when Ruderman et al. [13] described a case of patients with symptoms indicative of the metabolic syndromes—reduced insulin sensitivity, hypertension, T2DM, and hypertriglyceridemia—despite normal body mass index (BMI). Obesity of this type is defined as metabolically obese normal weight (MONW). Scientists did not develop a single set of diagnostic criteria for metabolic obesity in people with normal body weight. The aim of this study was to review the various principles for the diagnosis of MONW over the years.

## 2. Biological Mechanisms of MONW

Based on the research carried out so far in the MONW group (women and men, in different age groups and different ethnic groups), it can be concluded that the excessive accumulation of fat, mainly visceral, adversely affects the lipid profile [13,14,15], blood pressure [13,14], intensifies inflammatory and thrombotic processes [16], as well as oxidative stress [17]. On the other hand, in other studies in non-obese patients with an excessive accumulation of fat, no atherogenic lipid profile, differences in blood pressure values [18,19] or in the concentration of adipocytokines [14,15] were observed.

The central parts of the complex and still insufficiently recognized pathogenesis of MONW are the increased amount of visceral and subcutaneous fat in the abdominal area, insulin resistance and hyperinsulinemia, which are recognized as key disorders in MONW [14,19]. The increase in the mass of visceral adipose tissue causes increased lipolytic activity and the excess release of free fatty acids, which are accumulated in the liver and skeletal muscles. In the liver, increased very-low-density lipoprotein (VLDL) biosynthesis and reduced degradation, thereof, translate into an increase in the concentration of triglycerides in the blood plasma, and as a result of the action of lipoprotein lipase (LPL), cholesterol ester transfer protein (CEPT) and hepatic lipase (HL), LDL particles of high atherogenic potential are formed from VLDL particles. In addition, CETP-mediated multiplied lipid transport generates HDL particles of larger sizes. Hepatic insulin resistance is also manifested by increased glycogenolysis and gluconeogenesis, which increases endogenous glucose production and is associated with the development of non-alcoholic fatty liver disease (NAFLD) [20]. On the other hand, in skeletal muscles, the accumulation of biologically active lipids (long-chain acyl-CoA, diacylglycerols, ceramides) negatively affects the operation of the insulin pathway, inducing muscle insulin resistance, which is associated with impaired translocation of GLUT4 to the cell membrane and reduced transport of glucose to the interior myocytes, thus preventing glucose uptake [21]. This partially explains the complex relationships between obesity, insulin resistance, hyperglycemia and dyslipidemia.

Hypertrophic adipocytes are also a source of pro-inflammatory cytokines that enhance insulin resistance both in the fat cells themselves and in other tissues. Activated by inflammatory mediators (TNF-α, interleukin 1), nuclear factor kappa B (NF-kB) and c-Jun N-terminal kinase (JNK) pathways are the link between chronic inflammation and insulin resistance [22]. Obesity is accompanied by a subclinical chronic inflammation in which, in addition to activating pro-inflammatory signal transduction pathways, there is also an overexpression of pro-inflammatory cytokines in adipose tissue. Among the adipokines, whose activity may contribute to the development of metabolic disorders observed in MONW, the most frequently mentioned are resistin, leptin, adiponectin, TNF-α and IL-6 [17,23]. The pro-inflammatory and prothrombotic states are important components of the metabolic disorders associated with the excessive accumulation of adipose tissue, especially of the visceral type. The pro-inflammatory state is characterized by an increased concentration of cytokines such as TNF-α and IL-6, as well as an increased concentration of acute phase proteins—fibrinogen and CRP protein. The prothrombotic state is diagnosed on the basis of elevated levels of fibrinogen, PAI-1 and other coagulation factors. Increased biosynthesis of the above-mentioned cytokines by lipid-laden adipocytes causes not only tissue resistance to insulin but also pro-inflammatory state, endothelial dysfunction and disorders of coagulation and fibrinolysis.

There is evidence from experimental and clinical studies for a causal relationship between the amount of body fat and insulin resistance and the development and maintenance of elevated blood pressure. The increase in the prevalence of arterial hypertension especially concerns visceral obesity [24]. The etiological factors of arterial hypertension include: hemodynamic disorders accompanying obesity and an increase in peripheral vascular resistance associated with endothelial dysfunction, insulin resistance and the influence of adipokines released from adipose tissue [25].

The excess of energy substrates flowing into the cell in the form of free fatty acids and glucose causes the formation of an increased amount of acetyl-CoA and, thus, NADP in the mitochondria and, as a result, an increase in the biosynthesis of reactive oxygen species (ROS) and the development of oxidative stress [26].

**Figure 1:**
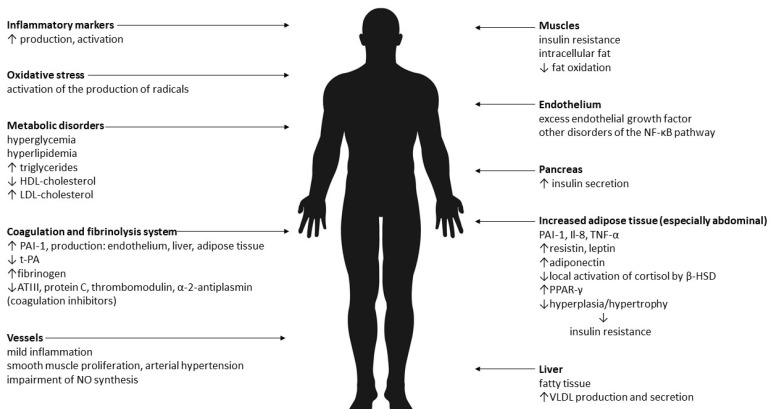
The effects of abdominal obesity and insulin resistance [13,14,15,16,17,18,19,20,21,22,23,24,25,26]. Legend: VLDL—very-low-density lipoprotein, NO—nitric oxide, PAI-1—plasminogen activator inhibitor-1, t-PA—tissue plasminogen activator, ATIII—antithrombin III, NF-κB—Il-8—interleukin-8, TNF-α—tumor necrosis factor α, β-HSD—beta- hydroxysteroid dehydrogenase, PPAR-γ—peroxisome proliferator-activated receptor gamma, HDL—high-density lipoprotein, LDL—low-density lipoprotein.

Therefore, it seems that the results of research on the pathogenesis of MONW to date are not unequivocal. The dominant causes are insulin resistance and abdominal obesity. It is believed that the cause of the changes is the increased mass of adipose tissue and its pro-inflammatory activity. Adipose tissue is an active endocrine and paracrine endocrine organ, and the secreted pro-inflammatory substances (adipokines) are an important link between excess body weight, insulin resistance, atherosclerosis and type 2 diabetes. In addition, there is oxidative stress. The effects of abdominal obesity and insulin resistance are summarized in Figure 1.

It is known that the occurrence of MONW is influenced by both environmental factors—lack of physical activity, unhealthy diet, smoking, alcohol consumption—and genetic factors. While comparing eating habits, controlled studies found that women with MONW consumed more saturated fat and less fiber than metabolically healthy women [27]. The effect of smoking was confirmed by Tilaki and Heidari [28]. Smoking was statistically significantly (*p* = 0.005) associated with the MONW phenotype in 170 men and women of Iranian origin. Research in the Korean population has shown that there is an association between the prevalence of MONW and moderate alcohol consumption, and a small amount of time for moderate-intensity physical activity [29]. Smoking and alcohol consumption as risk factors were confirmed in a meta-analysis by Wang et al. [30]. It is certain that genetic factors also have an influence on the occurrence of MONW. However, data on specific genes are quite limited. Li et al. [31] showed that CDKAL1 rs2206734 is associated with protection against the MONW phenotype. CDKAL1, which belongs to the methylthiotransferase family, increases translation efficiency and is widely expressed in metabolic tissues, including adipose tissue and pancreatic β cells. In turn, Park et al. [32] found links between the genes GCKR, ABCB11, CDKAL1, CDKN2B, NT5C2 and APOC1, and metabolic disorders in people with normal body weight.

## 3. Materials and Methods

### 3.1. Design

A literature review was conducted through the steps of Carnwell and Daly [33], which were (1) identifying the purpose of the literature, (2) exploring the articles using keywords that involve the scope of the literature, (3) organizing the results of the review and (4) determining the conclusion that will inform further studies.

### 3.2. Search Strategies

In this review, relevant articles were searched from electronic databases, including PubMed, ScienceDirect, Scopus and Google Scholar. The search process used the Boolean operator AND/OR on the combination of the following keywords: metabolically obese normal weight, metabolically abnormal but normal weight, metabolically unhealthy normal weight, normal weight obese, metabolically unhealthy non-obese, normal weight metabolically unhealthy, MONW, diagnostics.

### 3.3. Inclusion and Exclusion Criteria

Articles were included for review if they met inclusion criteria: (1) published in English within the publication date from 2002 to November 2021, (2) article types include original research and review articles, (3) fully accessed articles, of which a copy could be obtained by the authors. Articles were excluded if the main results were not in line with the purpose of this literature review.

## 4. Primary criteria for MONW

The author of the first MONW diagnostic criteria is Ruderman et al. [34], who in 1989 proposed a scoring system that assessed 22 features (Table 1) that were assigned a specific number of points. Obtaining at least 7 points was equivalent to the diagnosis of MONW.

This system had its drawbacks, requiring the performance of biochemical tests not routinely performed in healthy people (including uric acid concentration). For this reason, the search for much simpler and more accessible diagnostic criteria was started.

## 5. Anthropometric Indexes

Anthropometric measurements are one of the simplest methods by which obesity can be identified. The most commonly used and recommended by WHO and the International Obesity Task Force (IOTF) is BMI [35]. The BMI value in people with normal body weight is 18.5–24.9 kg/m^2^. Values from 25.0 to 29.9 kg/m^2^ are synonymous with overweight, while obesity is found in people with a BMI over 30 kg/m^2^ [3]. However, BMI has some limitations—it does not allow the assessment of body composition as it does not differentiate between lean and fat mass. Thus, a person with a normal BMI may have adequate body fat or excess body fat, which can be masked by normal BMI. In addition, the BMI cut-off points do not take into account differences in body proportions in different populations. For example, a study conducted on the basis of the Mauritian population, with the use of ethnically specific regression equations derived from the relationship BMI–percentage of body fat, suggests that the limits recommended by WHO seem to be valid only for Creole men (the limits of 24 and 29.7 kg m^2^, respectively, for overweight and obesity), but not for Creole women or for Indian men and women, whose BMI cut-offs are 3–4 units lower [36]. Moreover, several other studies questioned the WHO-recommended cut-off point for obesity: ≥31.5 kg/m^2^ in Lebanon [37], ≥22.2 kg/m^2^ for males and ≥24.5 kg/m^2^ for females for Ethiopian adults [38], ≥25.5, 28.7 and ≥26.2 kg/m^2^ for white, black and Hispanic women, respectively [39], or ≥24.9 kg/m^2^ for Brazilian women and ≥29.9 kg/m^2^ for Brazilian men [40]. Li et al., proposed cut-off point for Chinese: ≥22.5 and ≥25.9 (obesity and overweight, respectively) in men and ≥22.8 and ≥26.6 (obesity and overweight, respectively) in women [41].

The best anthropometric indicator of abdominal fat is the waist circumference (WC). Measurement is performed with a non-stretchable tape, at the midpoint between the lower rib and iliac crest [42]. In addition, it is the basis for other, more complex indicators, such as the waist-to-hip ratio (WHR). Hip circumference is measured at the widest part around the hip. WHR gained popularity in the late twentieth century when it was shown, alone or in combination with BMI, to be associated with increased risk of death, cardiovascular disease (CVD) and T2DM [43,44,45,46]. However, later studies provided evidence that waist circumference itself is more strongly associated with visceral fat, which represents the greatest health risk [47,48]. A similar and sometimes slightly stronger association with the risk of CVD or T2DM is shown by the waist-to-height ratio (WHtR) [49,50]. A likely explanation for this could be that short stature is associated with an increased risk of CVD [51]. In still growing children and adolescents, WHtR may be a more useful indicator of the classification of abdominal obesity than WC alone. However, in adults, WHtR is less useful as height is generally constant and the value can only be changed by changing the waist circumference [52].

Although all anthropometric measurements are simple and convenient methods, their accuracy in assessing obesity has been questioned because they are unable to distinguish lean mass from fat mass [53].

## 6. Adipose Tissue

The assessment of the fat depot is possible after measuring the body composition. This test allows for precise and accurate measurement of individual body components including muscle mass, lean mass and, most importantly, the percentage of adipose tissue (PBF,% BF), the knowledge of which, together with the BMI value, can be used as a screening tool [42,54].

Body composition can be assessed by various methods: computed tomography (CT), magnetic resonance imaging (MRI), dual-energy X-ray absorptiometry (DXA), electrical bioimpedance (BIA), hydrostatic plethysmography, isotope dilution techniques, skinfold method and air displacement plethysmography [55]. The most common methods are CT, DXA and BIA.

People with MONW are characterized by an increased content of adipose tissue—in particular, its visceral deposit [56]. Excessive abdominal fat storage is one of the causes of insulin resistance. Dvorak et al. [18] noticed that the increased amount of fat in the body of non-obese people (even by 2–3 kg) significantly reduced the sensitivity of tissues to insulin, while maintaining the correct BMI values. The mere information about the percentage of fat in the body is insufficient. It is important to establish a cut-off point that would indicate the existence of a metabolic disorder. Despite many studies, scientists do not agree on the PBF cut-off point above which obesity is diagnosed [57]. This also translates into different cut-off points used by scientists wishing to diagnose MONW. An important aspect in defining the cut-off point is the gender adjustment. In women, fat is stored mainly in the gluteofemoral depot (i.e., gynoid obesity), men accumulate fat in a visceral and abdominal depot (i.e., android obesity). The accumulation of subcutaneous fat in women is the result of the evolutionary preparation of the body for the development of pregnancy and breastfeeding. After the menopause, due to the lack of circulating estrogens, the distribution of adipose tissue changes–visceral fat accumulates [58]. This is confirmed by studies in which an adjusted decrease in visceral fat in postmenopausal women was observed after hormone therapy [59]. Estrogens enhance preadipocyte proliferation and differentiation into insulin-sensitive adipocytes and inhibit lipolysis, while androgens exert opposite functions. Moreover, the development and regulation of the fat depots in the female body are controlled by complex interactions between adipose tissue genes and ovarian hormones [60]. Gender differences are also noticed in adipogenesis. Male adipose tissue is characterized by more adipocyte hypertrophy, whereas females demonstrate more hyperplasia [61]. In conclusion, due to the fact that women physiologically have more body fat, the PBF cut-off points for MONW diagnostics among women should be higher. In premenopausal women, special attention should be paid to visceral fat content.

Katsuki et al. [14] initiated a series of studies of non-obese patients (BMI < 25 kg/m^2^) in terms of body composition. Using CT, they determined the area of adipose tissue. A deposit of more than 100 cm^2^ was tantamount to the diagnosis of MONW. The authors showed a significant correlation between visceral fat areas with serum triglycerides levels (r = 0.533, *p* < 0.02) and fasting serum levels of insulin (r = 0.503, *p* < 0.05) in subjects with MONW. The applied method of body composition analysis is characterized by high quantitative and qualitative accuracy, image resolution, and precision and sensitivity; unfortunately it is cost-consuming, requires trained operators and exposes the patient to ionizing radiation, which is a great difficulty in routine diagnostics [54,62].

De Lorenzo et al. [63] used the dual-energy X-ray absorptiometry method in their studies in non-obese people (BMI < 25 kg/m^2^). Their cut-off point for the PBF was 30%, which translated into the diagnosis of MONW in 28 out of 74 women (38%). Compared to CT, DXA is cheaper and, above all, exposes the patient to a much lower radiation dose. Radiation in DXA is on average 0.1–4.9 µSv compared to 2000–16,000 in CT [64].

Kim et al. [65] assumed the same cut-off point for women, while in the case of the surveyed men, values > 25% of PBF indicated MONW. The body composition analysis carried out using the BIA method showed that it allowed the diagnosis of metabolic obesity in 291 men out of 6534 (4.5%) and in 1281 women out of 5852 (21.9%). It is worth noting that the BIA method, despite its non-invasive nature and the low cost of the study, is met with criticism among scientists regarding the reliability of the obtained results. On the one hand, some studies report good accuracy of BIA [66,67] while others show poor results, especially among people with overweight or obesity through underestimation of the percentage of adipose tissue [68,69]. Probably, this is related with fluid distribution, resistive and volume properties among various body tissues [70]. BIA calculates the amount of fat-free mass (FFM) from total body water (TBW), assuming 73% of FFM is water in adults, therefore, a change in hydration leads to an underestimation of FFM [54]. The accuracy of the BIA method also depends on the number of electrical frequencies used—there are single-frequency (SF-BIA) and multifrequential (MF-BIA). The advantage of MF-BIA over SF-BIA is the possibility of extracting both intra- and extracellular fluid from the total body water [71].

Among the presented methods of body composition analysis, DXA is considered the “gold standard”. Studies have shown strong correlations between body composition parameters obtained by DXA and those obtained by CT [62,72,73,74,75]. Tewari et al. [62], moreover, showed a strong correlation between the DXA scans and the results obtained from MF-BIA. On the other hand, the analysis of the relationship between the CT and BIA results showed a weaker but still statistically significant correlation. It is worth noting that DXA is a three-compartment model of body composition analysis (fat, lean and bone components), while BIA is a two-compartment model (fat mass and fat-free mass) [71]. DXA, with high accuracy and precision, is commonly used as a reference method for developing and validating BIA equations [71]. Another important aspect in the analysis of body composition is the correct classification of subcutaneous and visceral fat. It is known that there is a difference in the attenuation of X-rays between these two types of adipose tissue. Mean absorption of subcutaneous adipose tissue, expressed in Hounsfield units (HU), is −190 to −30, in the case of visceral adipose tissue HU = −150 to 50—which confirms the advantage of methods using this radiation [76].

In a recent study, Correa et al. [77] defined MONW in women with PBF > 38.9% and in men > 25.5%. In the Latin population, the percentage of people diagnosed with MONW was 29.1% (2% for men and 46% for women, respectively).

Interesting research was performed by Tayefi et al. [78]. They examined a total of 2439 people with normal body weight (BMI < 25 kg/m^2^) aged 35–65 years. MONW diagnostics were based on two models. In model A, the PBF cut-off point was >25% for males and > 30% for females. In model B, the cut-off points depended on age:

20–39 years—>19% for men and >32% for women
40–59 years—>21% for men and >33% for women
60–79 years—>24% for men and >35% for women.

In model A, MONW was diagnosed in 38.5% of respondents, and according to model B—in 46.2%. Increasing the amount of adipose tissue with age is a physiological phenomenon, therefore, the adoption of different cut-off points depending on age seems to be the appropriate direction in the correct diagnosis of MONW.

Amato et al. [79], in 2010, proposed a new, gender-specific empirical and mathematical model called the visceral adiposity index (VAI). VAI is based on BMI, WC, triglycerides and HDL cholesterol:

$$\mathrm{Males}:\mathrm{VAI}=\left(\frac{\mathrm{WC}}{39.68+\left(1.88\times \mathrm{BMI}\right)}\right)\times \left(\frac{\mathrm{TyG}}{1.03}\right)\times \left(\frac{1.31}{\mathrm{HDL}}\right)$$

$$\mathrm{Females}:\mathrm{VAI}=\left(\frac{\mathrm{WC}}{36.58+\left(1.89\times \mathrm{BMI}\right)}\right)\times \left(\frac{\mathrm{TyG}}{0.81}\right)\times \left(\frac{1.52}{\mathrm{HDL}}\right)$$

The authors demonstrated a strong positive correlation between VAI and peripheral glucose consumption during the hyperinsulinemic euglycemia clamp [79] and the usefulness of the index in the assessment of possible visceral adipose tissue dysfunction (VAD) and cardiometabolic risk [80]. Subsequent studies have shown an association of VAI with blood pressure in both men and women [81], metabolic syndrome [82,83] and obstructive sleep apnea [84]. Moreover, it may constitute an independent risk factor for elevation of the Homeostatic Model Assessment (HOMA-IR) in both men and women [85] and a surrogate marker used to estimate the risk of metabolic disorders related to the accumulation of VAT, however, further studies are needed to determine the cut-off value [86]. Ferreira et al. [87], in their cross-sectional population study, showed that among the assessed obesity indices (VAI, BMI, WHR, WHtr, waist and neck circumference), VAI is the best predictor of the MONW phenotype in both sexes.

## 7. Biochemical Markers in MONW

One of the hallmarks of MONW is the decreased tissue sensitivity to insulin. The first to define MONW as the presence of insulin resistance (IO) was Dvorak et al. [18]. Among people with normal body weight (defined by the authors as BMI < 26.3 kg/m^2^), they determined tissue sensitivity to insulin using a metabolic clamp. The glucose consumption of 8 mg/min/kg lean body mass was adopted as the cut-off value for the diagnosis of IO. On this basis, MONW was identified in 13 out of 71 women (18%). The metabolic clamp, although considered the “gold standard”, is technically quite difficult, time-consuming and costly.

For the purpose of assessing insulin resistance, HOMA-IR was developed, which is based on measurements of fasting glucose and insulin levels. It is calculated from the formula [88]

$$\mathrm{HOMA}-\mathrm{IR}=\frac{\mathrm{fasting}\;\mathrm{insulin}\;\left(\frac{\mathrm{mU}}{\mathrm{mL}}\right)\times \;\mathrm{fasting}\;\mathrm{glucose}\;\left(\frac{\mathrm{mmol}}{L}\right)}{22.5}$$

Conus et al. [19] examined a total of 96 women with normal body weight (<25 kg/m^2^). After determining the concentration of fasting insulin and fasting glucose, they calculated the measurement of the HOMA-IR index with the cut-off value of 1.69. MONW was identified in 12 women (12.5%). Moreover, the authors concluded that among people with normal body weight, a significant decrease in insulin sensitivity of tissues was observed at lower values.

HOMA-IR has also been used by Lee at al. in 2011 [89]. This large cohort study studied 8987 nondiabetic people aged over 40 years. MONW was diagnosed in people with normal body weight (BMI < 23 kg/m^2^) with HOMA-IR value within the highest quartile. The prevalence of MONW was 14.2% for men and 12.9% for women.

Much interest in the diagnosis of metabolic disorders is focused on the triglycerides–glucose index (TyG), which is the product of fasting blood glucose and triglycerides [90]. It is calculated from the formula

TyG=natural logarith [fasting triglycerides (mg/dL)×fasting glucose (mg/dL)/2

Its usefulness as an indicator of insulin resistance has been confirmed in the Asian [90,91], Latin [92,93,94] and Caucasian [95,96] populations. The TyG index has been shown to be clinically useful in the identification of the metabolic syndrome [97,98]. Increased TyG index has been associated with the presence of risk factors for cardiovascular disease in healthy children and adolescents with a normal body weight [99]. Lee et al., who tested the role of the TyG index in identifying MONW subjects, proposed a TyG cut-off value for MONW ≥ 8.82 for men and ≥8.73 for women [90]. The team of Morales Gurrol et al. [100], also became interested in the use of this indicator in the diagnosis of MONW. In their research, 542 people were examined and as many as 354 (65.3%) people showed the MONW phenotype according to IDF criteria. The assumed cut-off point for the TyG was 4.68. The tested index showed a positive correlation with systolic blood pressure, diastolic blood pressure, fasting glucose and triglycerides, as well as a negative correlation with HDL-C concentrations. The authors suggested a strong relationship between insulin resistance (as measured by the TyG index) and the presence of the MONW phenotype.

Typically, in the identification of MONW, biochemical tests are carried out, mainly involving the measurement of glucose, insulin and triglyceride levels. Nevertheless, there is a trend in searching for new biochemical parameters that may indicate the above-mentioned phenotype of obesity.

The concentration of ferritin in the blood serum is a promising indicator. Apart from serving as a biomarker for the assessment of anemia, this protein is associated with insulin function and systemic inflammation. Serum ferritin concentration is elevated in the inflammatory environment and is associated with cardiometabolic disease, which is reflected in insulin resistance [101]. The incidence of the metabolic syndrome has been shown to increase with increasing serum iron levels. Moreover, several studies in western populations have shown that obesity, inflammation and the metabolic syndrome are most associated with increased serum ferritin levels [102,103,104,105]. Based on this information, Kim et al. [106] investigated the relationship between serum ferritin levels and MONW among 9411 healthy adult Koreans. The data for this study were from the fourth and fifth Korea National Health Examination and Nutrition Surveys (KNHANES). Combined diagnostic criteria were used to determine the metabolic status of the subjects, including the National Cholesterol Education Program Adult Treatment Panel (NCEPATP) III criteria, Wildman criteria and HOMA criteria. The detailed criteria included:

- BP ≥ 130/85 mmHg or use of antihypertensive medication,
- TG ≥ 150 mg/dL or use of lipid-lowering medication,
- FG ≥ 100 mg/dL or use of an oral hypoglycemic agent or insulin
- HDL cholesterol level <40 mg/dL in men and <50 mg/dL in women or use of medication for reduced HDL cholesterol level
- HOMA-IR greater than the 90th percentile in the nondiabetic general Korean population in KNHANES.

It was concluded that serum ferritin could be a clinical marker reflecting the risk and prevalence of MONW in adult Koreans with a normal BMI. The authors suggested the following serum ferritin cut-off values for the prediction of MONW: 46.87 ng/mL for women and 127.03 ng/mL for men. There are no identical studies for the European population in the literature.

In recent years, there has been a tendency to develop indicators using anthropometric measurements and biochemical tests. Kahn [107] proposed an indicator called “lipid accumulation product” (LAP), based on the combination of waist circumference measurements and fasting triglycerides. The developed formula assumes different waist circumference adjustments for men and women, which may result in problems in comparing the data yields.

$${\mathrm{LAP}}_{\mathrm{men}}=\;\mathrm{TG}\;\left(\mathrm{mmol}/L\right)\times \left(\mathrm{WC}\;\left(\mathrm{cm}\right)-65\right)$$

$${\mathrm{LAP}}_{\mathrm{women}}=\;\mathrm{TG}\;\left(\mathrm{mmol}/L\right)\times \left(\mathrm{WC}\;\left(\mathrm{cm}\right)-58\right)$$

Another disadvantage in using the index is the fact that it is below zero in women with WC < 58 cm and in men < 65 cm. LAP was intended by the authors to reflect both anatomical and physiological changes resulting from excessive lipid accumulation.

Studies found that LAP correlated better with variables of lipid risk, uric acid levels and heart rate compared to BMI. Another study by this author [108] showed that this index is much better in identifying adults with diabetes compared to BMI. In addition, LAP can be used to predict the risk of insulin resistance [109], cardiovascular disease [110] and the metabolic syndrome [83,111,112,113]. Du et al. [95] analyzed the ability of VAI and LAP to identify MONW using four diagnostic models. Both indicators have been shown to be strongly associated with this obesity phenotype, regardless of the definition model adopted. Moreover, the LAP and VAI were much superior to anthropometric indicators: BMI, WC, WHR and WHtR. These relationships were observed in both sexes and of all ages. Li et al. [114] assessed the usefulness of three indicators: LAP, VAI and TyG in the identification of the metabolic syndrome. Reliable predictive accuracy in the diagnosis of MetS has been demonstrated in both the ATPIII and IDF criteria, but it is worth noting that LAP turned out to be a better parameter than the other parameters used in the study. The same conclusions were drawn from their research by Shin et al. [115], who, in addition to the aforementioned indicators, also used WHtR for the diagnosis of MetS.

Lipid accumulation product became the foundation for the creation of another marker. Wakabayashi et al., in 2015 [116], proposed the cardiometabolic index (CMI), the components of which are the results of routine control tests.

$$\mathrm{CMI}=\frac{\mathrm{TG}\;}{\mathrm{HDLc}}\times \;\mathrm{WHtR}$$

The TG/HDL-C ratio used reflects small, dense, more atherogenic LDL particles, making it a better predictor of coronary artery disease than classical atherogenic indicators, including the LDL/HDL ratio. In their work, the authors emphasize that CMI may be a useful marker for distinguishing diabetes and suggest greater validity of using this indicator for predicting cardiovascular risk compared to WC or LAP [116]. Research by Liu et al. [117], conducted among 47683 participants, showed that 11% of them (5233 people) met the MONW phenotype criteria, according to the NCEP-ATPIII guideline that specifically applied for Asians. These individuals were characterized by higher values of the CMI, systolic blood pressure (SBP), diastolic blood pressure (DBP), total cholesterol (TC), FG, WC, BMI, TyG, LDL-C, uric acid, alanine transaminase (ALT) levels and creatinine, and lower HDL-C than their MONW counterparts (*p* < 0.001). This study assessed the usefulness of CMI in identifying patients with MONW and comparing it with traditional obesity rates. CMI was shown to be positively and independently associated with the presence of the MONW phenotype. Moreover, it was confirmed that CMI is a better screening tool for MONW patients compared to BMI and WC. Li [118] showed that both LAP and CMI are strongly associated with abnormal fasting glucose levels in Chinese people without obesity.

## 8. MetS Criteria

Some scientists use diagnostic criteria for classic metabolic syndrome (MetS) in their research on MONW, according to the criteria of the National Cholesterol Education Program Adult Treatment Panel III (NCEPATP III) or proposed by the International Diabetes Federation (IDF). The coexistence of at least three of the five disorders mentioned is synonymous with the occurrence of MetS (Table 2).

In the case of the criteria proposed by the IDF, the percentage of people diagnosed with MONW ranges from 9.46% in the Serbian population [121] and 12% in the Finnish population [122], to 14.3% in the Korean population [123]. On the other hand, Lu, based on the criteria proposed by NCEPATP III (but with a BMI limited to 24 kg/m^2^), diagnosed MONW in 12.67% of Asians [124]. This discrepancy in the results prompted scientists to look for further diagnostic criteria and cut-off points for a given population.

Wildman et al., based on the MetS criteria, proposed his own set of criteria (the presence of at least two anomalies proves MONW):

- BP ≥ 130/85 mmHg or antihypertensive medication use
- Fasting TG level ≥ 150 mg/dL
- HDL-C level M: <40 mg/dL, W: <50 mg/dL or lipid-lowering medication use
- FG level ≥ 100 mg/dL or antidiabetic medication use
- HOMA-IR > 5.13 (i.e., the 90th percentile).

The prevalence of MONW depending on age was as follows:

20–34 years—10.3%
35–49 years—16.9%
50–64 years—41.7%
65–79 years—54.7%
>80 years—56.2%.

Rotar et al. [125], in turn, abandoned the calculation of the HOMA-IR index, adding the waist circumference measurement (M: >102 cm, W: >88 cm), a cut-off point for BMI < 30 kg/m^2^. Based on these criteria, MONW (or as the authors define it—MUNO) was diagnosed in 4762 people (34.4%), of which 2055 (35.4%) were women and 2707 (32.5%) were men. Such a high result is probably the result of adopting such a high cut-off point for BMI.

Luo et al. [126], in a study in the Chinese population, defined MetS according to the Joint Committee for Developing Chinese Guidelines on the prevention and treatment of dyslipidemia in adults (JCDCG) definition. MetS was defined as the presence of ≥3 of the following abnormalities:

- waist circumference > 90 cm for men and >85 cm for women;
- TG ≥ 1.70 mmol/L or specific treatment for lipid abnormality;
- HDL-C < 1.04 mmol/L or specific treatment for lipid abnormality;
- blood pressure ≥ 130/85 mmHg or known treatment for hypertension;
- FG ≥ 6.1 mmol/L and/or 2 h plasma glucose ≥ 7.8 mmol/L and/or diabetes mellitus having been diagnosed and currently receiving therapy.

The cut-off points for PBF were, respectively, <25% for men and <35% for women. According to this criteria, MONW was diagnosed in 90 out of 1186 men (7.6%) and in 95 out of 1578 women (6.0%).

## 9. Conclusions

The cases of metabolic disorders in people with normal body weight, described in the 1980s by Ruderman [13], gave rise to research on a new phenomenon—metabolic obesity in people with normal body weight (MONW). The primary diagnostic criteria were complex and required the use of tests not routinely used in healthy subjects. For this reason, subsequent studies have attempted to simplify the current criteria and develop new indicators specific to MONW.

The key phrase “correct body weight” refers to BMI, which, according to WHO and IOTF recommendations, should be in the range of 18.5–24.9 kg/m^2^ [3]. Nevertheless, many scientists adopt different limit values e.g., ≥23 kg/m^2^ [89] or ≤27 kg/m^2^ [127].

Diagnostics of MONW should begin with anthropometric tests—in particular, waist circumference and body composition testing. The key is to determine the percentage of adipose tissue and its distribution in the body. Particular attention should be paid to visceral fat, which is significantly more metabolically and hormonally active compared to its subcutaneous counterpart. When analyzing the fat deposit, it is reasonable to take into account the adjustment for the age and gender of the test person. There are many methods of body composition analysis, from computed tomography, through dual-energy X-ray absorptiometry, to electrical bioimpedance. The “gold standard” is DXA, which is more precise than BIA and has a lower radiation dose than CT.

People with MONW, in addition to increased fatty tissue deposit, are also accompanied by metabolic disorders, including, in particular, carbohydrate metabolism and insulin resistance. Currently, there is a trend in the diagnosis of MONW using the diagnostic criteria of the classical metabolic syndrome. Nevertheless, scientists are looking for further disorders that may indicate abnormalities (e.g., serum ferritin concentration [106]). There is also great potential in the indicators using anthropometric and biochemical tests—lipid accumulation product (LAP) [107] or cardiometabolic index (CMI) [116].

In summary, MONW is undoubtedly a growing problem that should be the focus of further research. Due to the fact that it is a disease that does not show phenotypic signs, screening tests should be carried out, mainly including body composition analysis among young, theoretically healthy people. This will allow for early detection of MONW and appropriate reactions before the occurrence of undesirable consequences—including atherosclerosis or coronary artery disease.

## Figures and Tables

**Table 1:** A point scale to identify people with MONW [34].

Points	Symptoms
1	triglycerides level > 100–150 mg/dL blood presure 125–140/85–90 mmHg weight gain: >4 after 18 years for women and 21 years for men BMI: 23–25 kg/m^2^ waist: 71.1–76.2 for women and 86.3–91.4 for men ethnicity: black women, Japanese-Americans, Latinos, Melanesians, Polynesians, New Zealand Maoris
2	impaired fasting glucose (110–125 mg/dL) triglycerides level > 150 mg/dL blood presure > 140/90 mmHg essential hypertension (under age 60 years) premature coronary heart disease (under age 60 years) low birth weight (<2.5 kg) inactivity (<90 min aerobic exercise/week) weight gain: >8 after 18 years for women and 21 years for men BMI: 25–27 kg/m^2^ waist: >76.2 for women and >91.4 for men uric acid (>8 mg/dL) ethnicity: Indians, Australian aborigines, Micronesians, Naruans
3	gestational diabetes triglycerides level > 150 mg/dL and HDL cholesterol < 35 mg/dL type 2 diabetes mellitus or impaired glucose tolerance hypertriglyceridemia weight gain: >12 after 18 years for women and 21 years for men premature coronary heart disease (under age 60 years) ethnicity: some American Indian tribes
4	type 2 diabetes mellitus impaired glucose tolerance polycystic ovaries

**Table 2:** Diagnostic criteria for the Metabolic Syndrome.

Measure	NCEPATP III [119]	IDF [120]
WC	>102 cm for men >88 for women	≥94 cm for men ≥80 cm for women *
TG	>1.7 mmol/L	>1.7 mmol/L or treating hypertriglyceridemia
High-density lipoprotein (HDL) concentration	<1.3 mmol/L for men <1.03 mmol/L for women	<1.0 mmol/L for men <1.3 mmol/L for women or treating said lipid disorder
BP	>130/80 mm Hg	≥130 mm Hg systolic or ≥85 mm Hg diastolic or treatment of previously diagnosed arterial hypertension;
FG	>6.1 mmol/L	≥5.6 mmol/L or drug treatment of type 2 diabetes

Legend: WC—waist circumference; TG—concentration of triglycerides; BP—blood pressure; FG—fasting glucose; * in the European population.

## Data Availability

We exclude this statement because this study did not report any data.
